# Secretion of Blood Instead of Milk

**Published:** 1890-09

**Authors:** 


SECRETION OF BLOOD INSTEAD OF MILK.—Dr. Habergritz reports a case of the secretion of blood in the breasts. The patient was a young married woman who, when she had been pregnant with her first child about six months, consulted Dr. Habergritz as to whether the fœtus was alive. He noticed some blood-stains on her linen in the neighborhood of the breasts, and on examination found that drops of pure blood could be expressed. The patient said that the bleeding had begun when she was five months pregnant, and she did not know that it was an unusual occurrence, and therefore had not mentioned it. During the rest of the pregnancy the phenomenon continued, and the patient suffered besides from two or three attacks of epistaxis. Two days before labor came on the bleeding ceased, but it reappeared in increased amount the day after. The patient was very anxious to nurse the child, but as it drew nothing but blood this had to be put a stop to. On the seventh day the color of the secretion began to change, and by the eighth it had all the characters of ordinary colostrum. The child was then allowed to take the breast, and nothing further abnormal was observed. It should be mentioned that the woman was perfectly healthy; there were no traces of gout, hæmorrhoids, cancer, or of hemorrhagic diathesis.—*The Lancet.*

